# Fasciculoventricular accessory pathway masked extensive atrioventricular conduction system disease in a patient with PRKAG2 syndrome

**DOI:** 10.1111/anec.13134

**Published:** 2024-06-27

**Authors:** Fuhan Gong, Long Yang, Qifang Liu

**Affiliations:** ^1^ Department of Cardiology Tongren Municipal People's Hospital Tongren Guizhou China; ^2^ Department of Cardiology Guizhou Provincial People's Hospital Guiyang China

**Keywords:** 12‐lead ECG, atrial flutter, conduction block, fasciculoventricular pathway, preexcitation

## Abstract

A 23‐year‐old male with a history of ventricular pre‐excitation and atrial flutter presented for evaluation after recurrent syncope. The possible mechanism of syncope erroneously attributed to pre‐excited atrial flutter with fast heart rates in the first hospitalization. The patient was found to have advanced heart block and PRKAG2 genetic mutation in the second hospitalization. The genetic findings and clinical features are consistent with PRKAG2 syndrome (PS). PS is a rare, autosomal dominant inherited disease, characterized by ventricular pre‐excitation, supraventricular tachycardia, and cardiac hypertrophy. It is frequently followed by atrial–fibrillation‐induced ventricular fibrillation and advanced heart blocks. An accurate differential diagnosis of syncope is important because of the different arrhythmic features and clinical course of PS.

## Graphical abstract

The ECG demonstrates preexcitation and atrial flutter. The stripe of the Holter recording shows sinus rhythm and frequent premature atrial complexes with extensive conduction system disease. The patient with unexplained left ventricular hypertrophy and fasciculoventricular pathways was diagnosed as PRKAG2 syndrome by gene analysis.

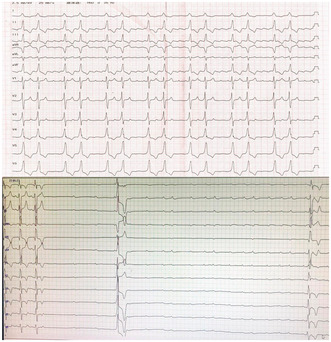

## 1 INTRODUCTION

PRKAG2 syndrome (PS) is a rare, autosomal dominant inherited disease, clinically characterized by ventricular pre‐excitation, progressive conduction system disease, and ventricular hypertrophy (Murphy et al., 2005). The syndrome is caused by mutations in the gene encoding for 5'‐adenosine monophosphate‐activated protein kinase (AMPK), specifically its γ2 regulatory subunit (PRKAG2). AMPK modulates glucose uptake and glycolysis in myocytes and increases intracellular glycogen deposition, resulting in cardiac hypertrophy and arrhythmias (Cao et al., 2017). Several malignant arrhythmias are the most common manifestation, from a sudden onset high‐degree AV block to ventricular fibrillation which can be linked to atrial fibrillation and ventricular pre‐excitation (Lopez‐Sainz et al., 2020). Sudden cardiac death (SCD) is a severe complication that occurs in 27% of PS patients, therefore, early identification and monitoring are very important for patient management (Ahamed et al., 2020).

## 2 CASE PRESENTATION

**FIGURE 1:**
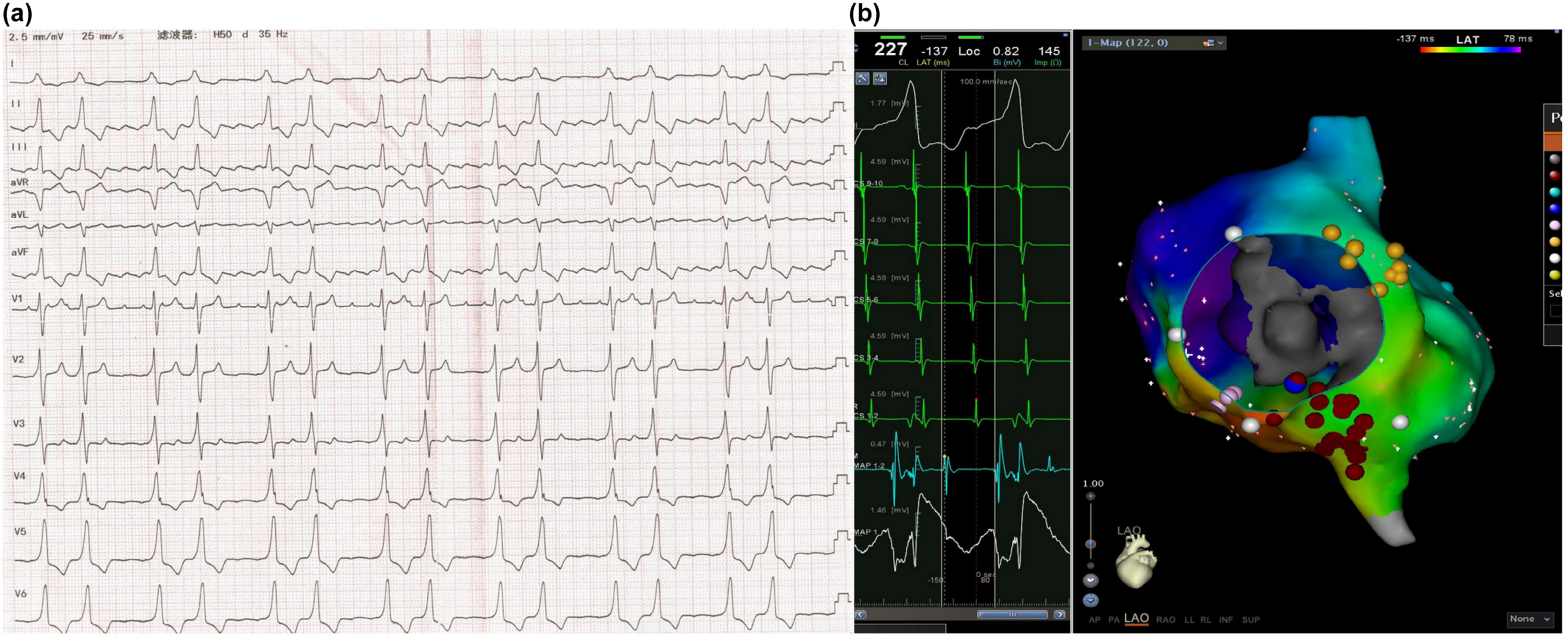
(a) Baseline ECG showing preexcitation and atrial flutter. (b) Activation map shows typical atrial flutter.

A 23‐year‐old man was referred to our hospital for palpitation and a history of syncope. Laboratory examination revealed that myocardial enzymes and troponin I level were normal, and his serum potassium level was 4.3 mmol/L. Baseline 12‐lead electrocardiogram (ECG) showed atrial flutter (AF) and ventricular preexcitation (Figure 1a). ECG showed unexplained left ventricular hypertrophy and reduced left ventricle ejection fraction (48%) (Figure S1).

**FIGURE 2:**
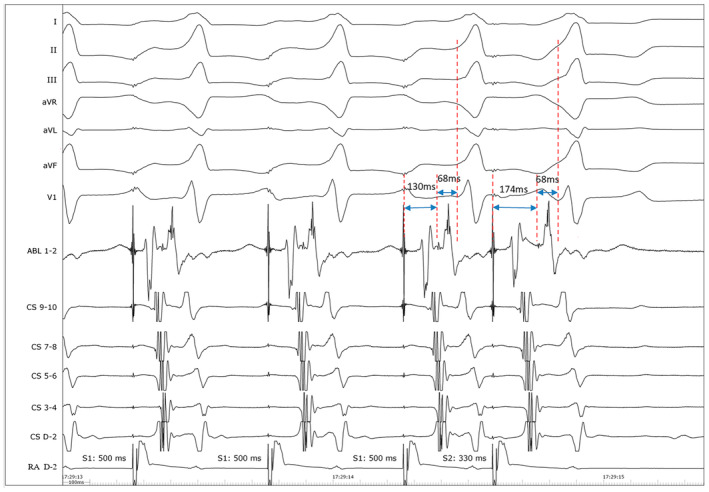
Following an atrial premature beat, there was prolongation of the AH from 130 to 174 ms, without change in HV intervals (68 ms) and QRS complex morphology and duration.

Electroanatomic mapping was performed using CARTO (Biosense Webster). Activation mapping was performed using a 3.5‐mm irrigated tip quadripolar ablation catheter (ThermoCool, Biosense Webster) and showed typical atrial flutter. Successful ablation was achieved by radiofrequency application in the cavotricuspid isthmus (Figure 1b). Baseline electrophysiologic examination was performed in sinus rhythm and the HV interval was at 68 ms. Programmed atrial extrastimuli cause progressive prolongation of the AH interval but no change in the HV interval, the degree of preexcitation, QRS configuration, and ventricular activation sequence (Figure 2). Based on these findings, the presence of a fasciculoventricular (FV) accessory pathway (AP) was confirmed. Furthermore, atrial premature depolarization at 310 ms produces a block in the fasciculoventricular pathway without anterograde conduction over the AV nodal pathway. Neither rapid antegrade conduction nor inducible tachycardias were observed by isoproterenol and programmed electrical stimulation. No ablation was attempted and the patient was discharged (Figure S2).

**FIGURE 3:**
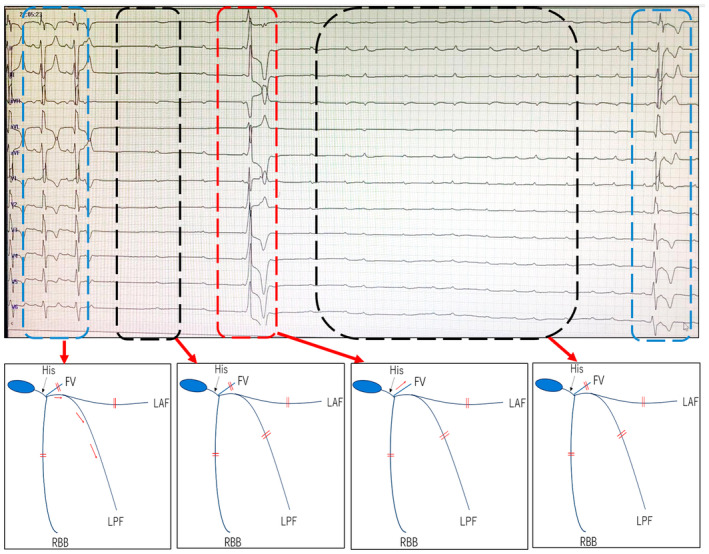
The ECG demonstrates sinus rhythm and frequent premature atrial complexes with complete AV block and intermittent LPF or FV AP conduction. The lower panel showing model diagram of the possible mechanism of conduction pattern. The double red line representing conduction block. The first two QRS morphology (blue dotted box) was characterized by a pattern of RBB and LAF block with normal PR interval, indicating LPF conduction. The first black dotted box showed complete AV block, indicating trifascicular and FV AP conduction block. The QRS morphology (red dotted box) was characterized by ventricular preexcitation, indicating FV AP conduction resumption. The second black dotted box showed complete AV block, indicating FV AP conduction block again. FV, fasciculoventricular accessory pathway; LAF, left posterior fascicular; LPF, left posterior fascicular; RBB, right branch bundle.

In a month, the patient experienced many episodes of syncope again during which telemetry documented rapid atrial arrhythmia and intermittent third‐degree atrioventricular block without junctional escape beat (Figure S3). Twenty‐four‐hour Holter monitoring showed sinus rhythm and frequent premature atrial complexes with alternative left posterior fascicular (LPF) and FV AP conduction, followed by complete atrioventricular block (Figure 3). These findings indicated extensive conduction system disease in all three fascicles: the right bundle fascicle block (RBBB), left posterior fascicular block (LPFB), and left anterior fascicular block (LAFB). Uncommonly, intermittent conduction over FV AP was observed in the patient. Direct sequencing of the PRKAG2 gene revealed the missense mutation in exon 7 (c.905G>A and Arg302Gln) (Figure S4). Patients with the PRKAG2 mutation are known to be at high risk for dying from atrial–fibrillation‐induced ventricular fibrillation and from complete AV block (Porto et al., 2016). ICD implantation was performed in the patient for secondary prevention based on sudden cardiac arrest.

## 3 DISCUSSION

PRKAG2 mutations typically cause PS which presents a familial syndrome of ventricular preexcitation, conduction defects, and cardiac hypertrophy (Jhaveri et al., 2019). On the first hospitalization, the FV AP with antegrade effective refractory period 310 ms masked extensive AV conduction system disease. Though evaluating the antegrade effective refractory period of the bypass tract may provide information relative to the cause of syncope, the overwhelming evidence suggests that one cannot use the antegrade effective refractory period measurements to predict patients at risk for development of atrial–fibrillation‐induced ventricular fibrillation. Therefore, the possible cause of the patient's syncope subjectively attributed to pre‐excited atrial flutter with fast heart rates.

For the second hospitalization, 24‐h Holter ECG monitoring showed the alternative conduction of LPF and FV AP followed by complete AV block. The mechanism of the patient's syncope was related to third‐degree atrioventricular block without an escape rhythm. Genetic analysis was performed in the patient, identifying a missense pathogenic variant in the PRKAG2 gene. Patients with the PRKAG2 mutation are known to be at high risk for complete AV block without a reliable escape rhythm (Sternick et al., 2011). In our case, the patient is wrongly diagnosed as idiopathic hypertrophic cardiomyopathy and fasciculoventricular pathways because deficiency of genetic analysis in his first hospitalization. Therefore, genetic assessment should be analyzed to correctly identify these patients because of their ominous prognosis due to the high incidences of malignant arrhythmias and SCD.

Despite PRKAG2 mutation having a high incidence of complete atrioventricular block, the exact site of the block was not described in previous studies. To our knowledge, we first described RBBB, LAFB with alternating LPF and FV AP block was responsible for intermittent third‐degree atrioventricular block in this case. Furthermore, HV interval was 68 ms in the patient, It is reasonable to infer that this significantly long HV interval might suggest pathological slow conduction in the fasciculoventricular pathway and conduction system. In brief, patients with the PRKAG2 mutation are known to be at high risk for syncope from complete AV block and atrial–fibrillation‐induced ventricular fibrillation, this electrophysiological phenomenon for patients with unexplained left ventricular hypertrophy and ventricular preexcitation should be acknowledged to avoid misdiagnosis and inappropriate therapy for syncope.

## AUTHOR CONTRIBUTIONS

Fuhan Gong wrote the manuscript, Long Yang processed the data and performed analysis, Qifang Liu designed and dericted the project. Ethics statement: The study was approved by the research ethics committee of Guizhou provincial people’s hospital.

## FUNDING INFORMATION

This study was supported by the Guizhou Provincial Science and Technology Agency Project (Qian Ke He Basic‐ZK [2022] General 255).

## CONFLICT OF INTEREST STATEMENT

The authors declare no conflict of interest.

## INSTITUTIONAL REVIEW BOARD STATEMENT

The study was approved by the Research Ethics Committee of the GuiZhou Provincial People's Hospital.

## INFORMED CONSENT STATEMENT

Informed consent was obtained.

## Supporting information


Figure S1
Figure S2
Figure S3
Figure S4

Data S1

## Data Availability

The data that support the findings of this study are available in this published article and its supplementary information files.
