# Supplementary material for: Fasciculoventricular accessory pathway masked extensive atrioventricular conduction system disease in a patient with PRKAG2 syndrome
Source: Ann Noninvasive Electrocardiol. 2024 Jun 27;29(4):e13134. doi: 10.1111/anec.13134 (PMC11211205; doi:10.1111/anec.13134)
Supplement: Supplementary file 1 — Figure S1 Figure S2 Figure S3 Figure S4 [file ANEC-29-e13134-s001.docx]

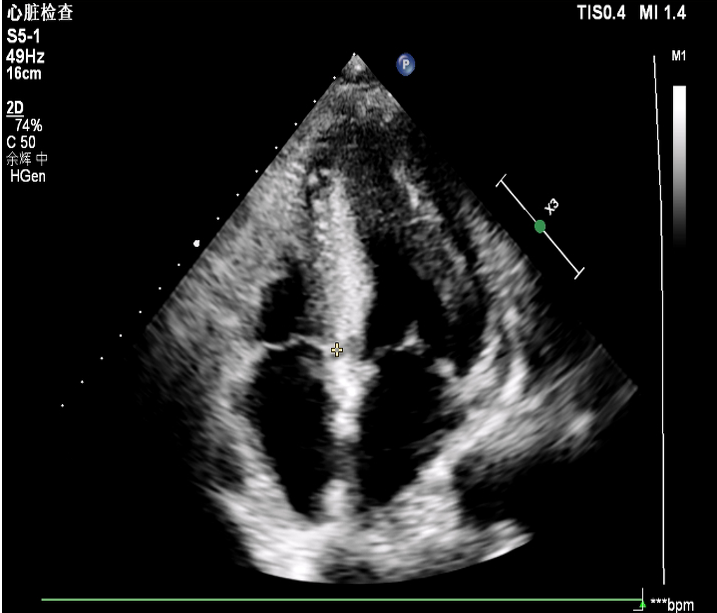


Figure S1：Echocardiography showed myocardial hypertrophy.


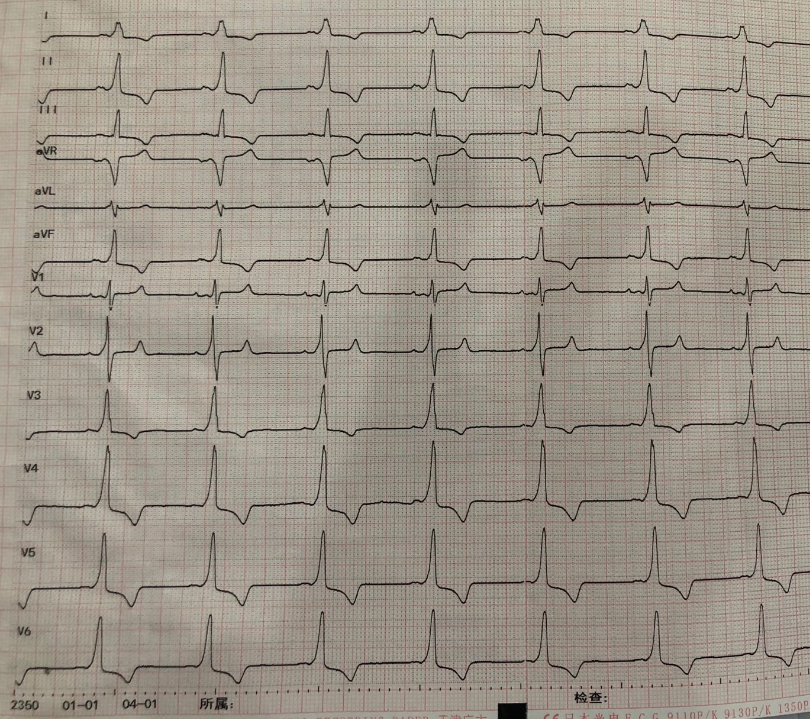


Figure S2: The ECG demonstrates sinus rhythm with fasciculoventricular accessory pathway after the operation


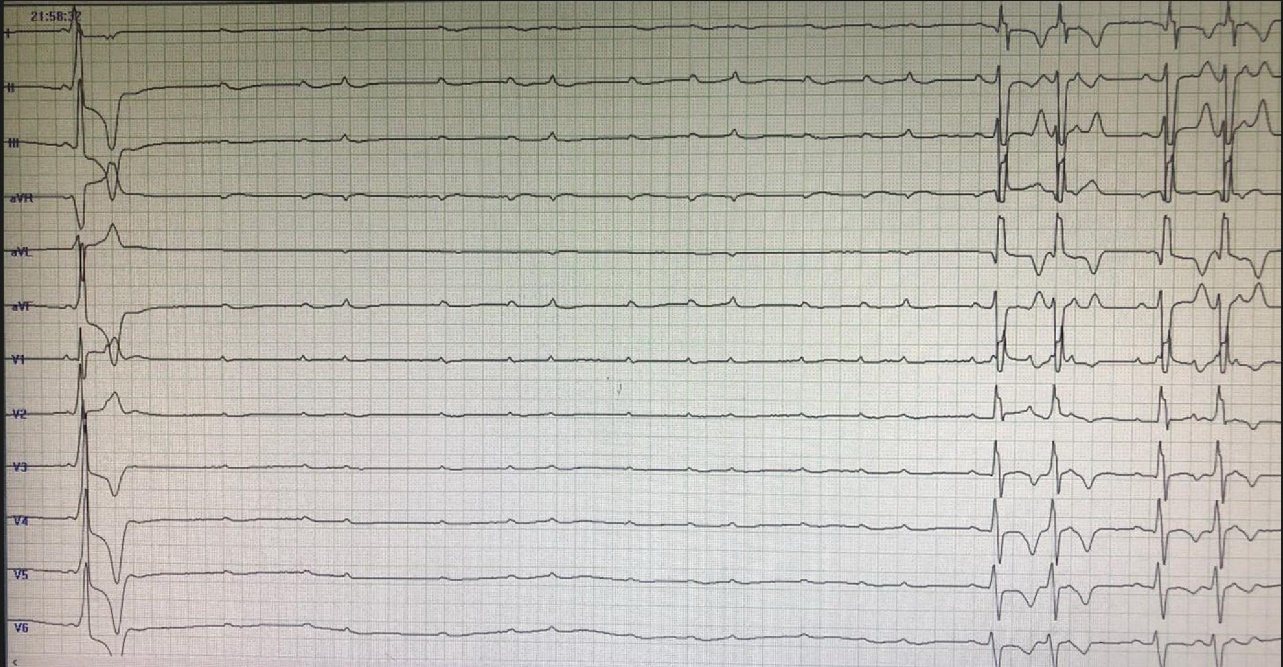


Figure S3: The ECG demonstrates sinus rhythm and frequent premature atrial complexes with complete AV block in a month after the operation


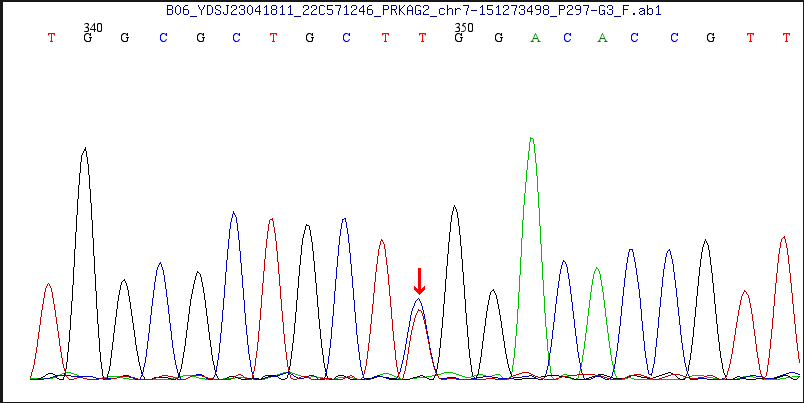


Figure S4: Gene analysis demonstrated PRKAG2: c.905G>A and Arg302Gln
