# Supplementary figures and images for: Fasciculoventricular accessory pathway masked extensive atrioventricular conduction system disease in a patient with PRKAG2 syndrome
Source: Ann Noninvasive Electrocardiol. 2024 Jun 27;29(4):e13134. doi: 10.1111/anec.13134 (PMC11211205; doi:10.1111/anec.13134)

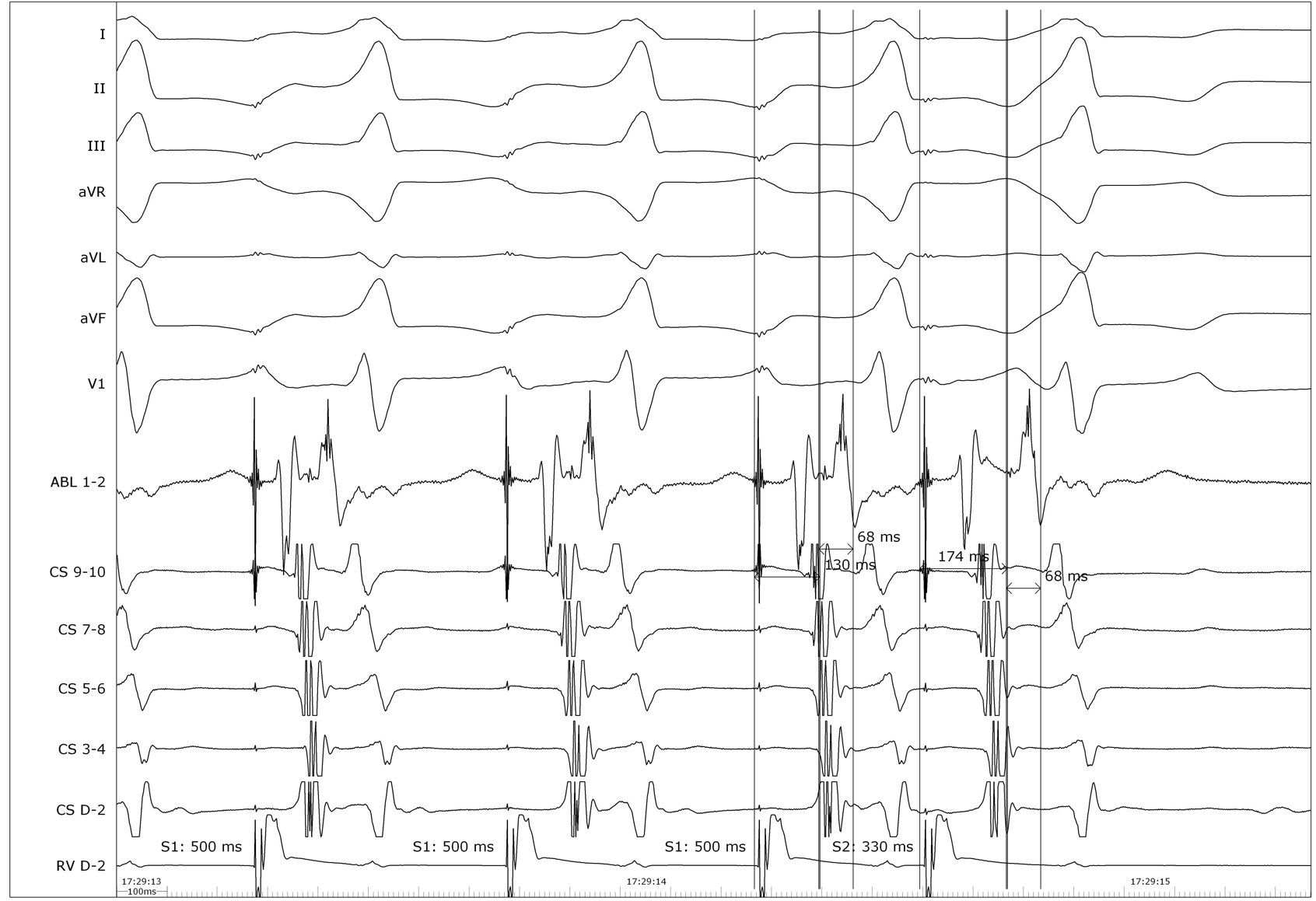

Supplement: Supplementary file 2 — Data S1 [file ANEC-29-e13134-s002.png]
